# Acute Limb Ischemia Caused by Mural Thrombus from an Abdominal Aortic Aneurysm Treated with Thrombectomy and Endovascular Aneurysm Repair: A Case Report

**DOI:** 10.3400/avd.cr.26-00005

**Published:** 2026-05-30

**Authors:** Ami Kojima, Yutaka Matsubara, Takuma Kai, Kazuomi Iwasa, Tadashi Furuyama

**Affiliations:** Department of Vascular Surgery, NHO Kyushu Medical Center, Fukuoka, Fukuoka, Japan

**Keywords:** abdominal aortic aneurysm, mural thrombus, acute limb ischemia

## Abstract

Acute limb ischemia (ALI) attributable to mural thrombus in an abdominal aortic aneurysm (AAA) is a clinically relevant complication owing to its high mortality risk. We encountered a patient with a floating thrombus in an AAA, which caused ALI. Computed tomography revealed an AAA with irregular mural thrombi, along with left popliteal artery occlusion. We performed urgent thrombectomy followed by elective endovascular aneurysm repair without any major complications. The keys to this successful outcome were earlier extremity reperfusion and less invasive aortic repair.

## Introduction

Peripheral embolization due to mural thrombus in an abdominal aortic aneurysm (AAA) is a clinically relevant complication.^1)^ Microvascular embolization, such as cholesterol embolization, is sometimes observed in patients with AAA; however, a few cases of acute limb ischemia (ALI) have also been reported.^2–4)^ Mortality after AAA-induced ALI is high.^1)^ Besides the most common complication of rupture, ALI is also a fatal AAA complication that requires rapid and appropriate interventions.^1–4)^ In this report, we present a patient with a floating thrombus in an AAA that caused acute popliteal artery occlusion and was successfully treated with thrombectomy and endovascular aneurysm repair (EVAR).

## Case Report

A 66-year-old man with a history of hypertension and a current smoking habit was admitted to the emergency department owing to a sudden onset of left lower-limb pain and coldness. His left lower leg and bilateral toes were pale with cyanosis. The popliteal arteries were normally palpable on both sides; however, the left dorsalis pedis and the posterior tibial arteries were not detectable by Doppler. This indicated microembolization involving the bilateral toes and left below-knee arterial occlusion. Sensory disturbance was also present. Laboratory tests revealed marked elevations in white blood cells (21000/μL) and D-dimer (50.5 μg/mL) but not creatine kinase (162 U/L).

**Fig. 1:**
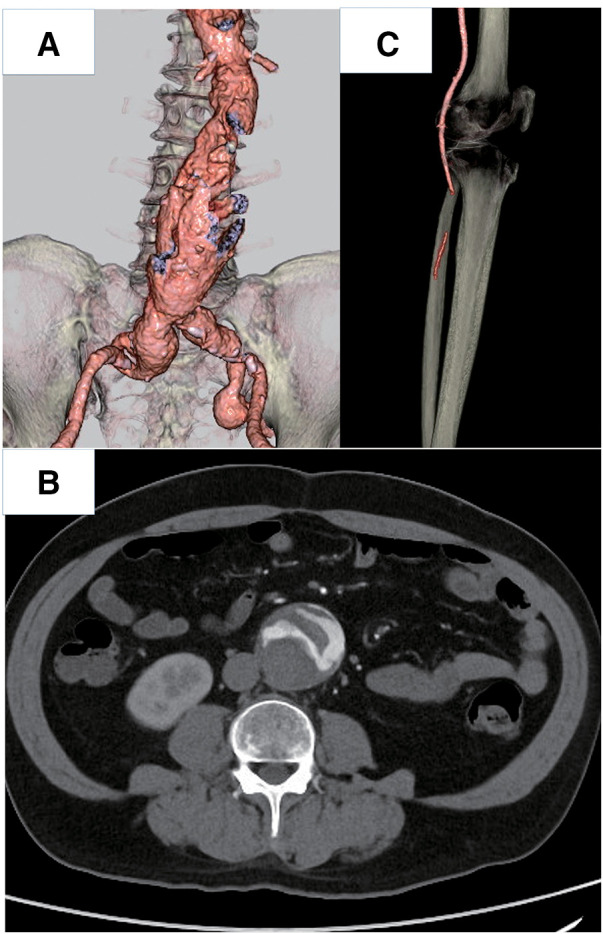
CT scans upon admission. (**A**) CT angiography of an AAA. (**B**) An axial image showing heterogeneous mural thrombus in an AAA. (**C**) CT angiography of the left limb. Arteries below the knee were not detected. CT: computed tomography; AAA: abdominal aortic aneurysm

Contrast-enhanced computed tomography (CT) demonstrated an AAA with irregular mural thrombi extending from the AAA sac to the aortic bifurcation (**Fig. 1A** and **1B**), along with complete occlusion of the left below-knee popliteal artery (**Fig. 1C**); the AAA diameter was 50 mm. Thus, the patient was diagnosed with Rutherford grade IIb ALI. Emergency thrombectomy for the left popliteal artery was performed the same day. The left common femoral artery was cut, and thrombectomy was performed with a 3-Fr Fogarty catheter. Angiography revealed straight-line but slow flow to the posterior tibial artery. We did not identify any other areas of distal embolization on final angiography, but blood flow was slow because of spasm. The patient did not develop calf swelling after revascularization. We provided hydration at 1.5 mL/kg/h, but specific re-perfusion injury management, including fasciotomy, was not required.

**Fig. 2:**
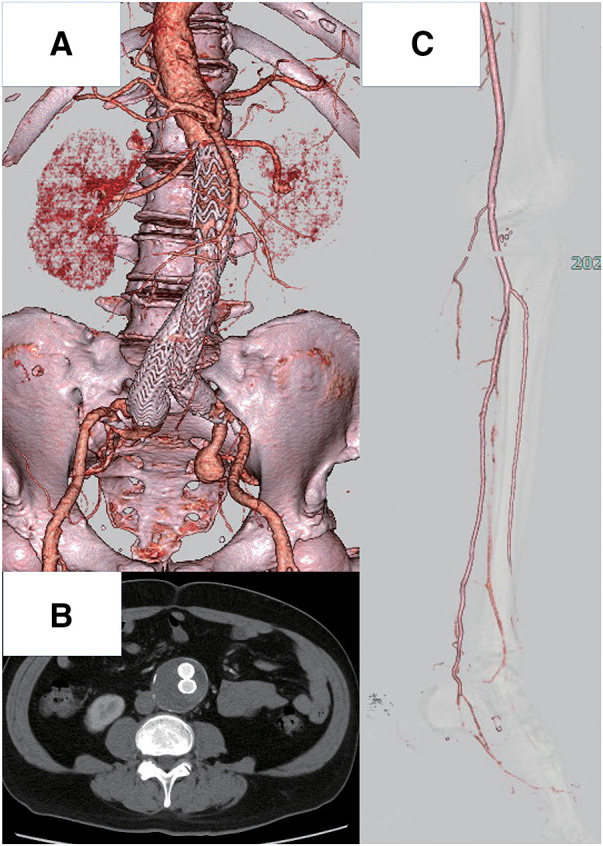
Postoperative CT scans. (**A**) CT angiography after EVAR. (**B**) An axial image at the same level as that in **Fig. 1B**. (**C**) CT angiography of the left limb. Arteries below the knee can be detected. CT: computed tomography; EVAR: endovascular aneurysm repair

On postoperative day 2, creatine kinase was maximally elevated (40104 U/L), and minor gangrene was observed in the bilateral toes, which were cyanotic at admission. Creatine kinase level was decreased to 287 U/L without acute renal failure during 2 weeks of observation after thrombectomy. The patient was free from any other postoperative complications after thrombectomy. Subsequently, EVAR was performed (**Fig. 2A**). We used a Gore Excluder stent-graft (main body, CXT361414; right leg, PLC271400; left leg, PLC231400; W. L. Gore & Associates, Flagstaff, AZ, USA). We exposed the bilateral common and superficial femoral arteries, and the bilateral superficial femoral arteries were clamped during the procedure to prevent distal embolization. We did not clamp the deep femoral arteries to maintain blood flow to the limbs. Postoperative CT scans showed a patent abdominal aorta without any thrombus (**Fig. 2B** and **2C**). Dry gangrene developed on the tip of the left great toe and on the right second and third toes, requiring minor amputations without any further revascularization. The wounds were closed primarily, and postoperative healing was satisfactory.

## Discussion

This paper reports a case of ALI due to an AAA mural thrombus. Most previous reports demonstrated aortoiliac occlusion,^2–4)^ whereas our patient developed limb embolization. The AAA had unstable plaques with heterogeneous enhancement (**Fig. 1**), and urgent thrombectomy and elective EVAR were performed. Although minor amputations were required, the patient was discharged without any major complications.

This case demonstrates how to prevent secondary embolization due to mural thrombus in an AAA. Open aortic repair is the first choice for AAA, with superior long-term outcomes compared to those of EVAR.^5)^ Furthermore, further thrombosis can be prevented by distal artery clamping and washing the clots out. Conversely, the disadvantages of open repair include its invasiveness. Immediately after thrombectomy, patients usually undergo heparinization,^6,7)^ which increases blood loss, thereby requiring additional transfusion. If the patient were to not develop ALI, we considered open repair; however, to minimize invasiveness, we chose EVAR. To decrease the risk of potential thromboembolization, we performed EVAR with superficial femoral artery clamping during the procedure. This patient had a thrombus only in the AAA; therefore, we determined that the risk of visceral embolization was as usual. The patient did not develop any post-EVAR complications. Postoperative CT scans showed complete exclusion of the intraluminal thrombus (**Fig. 2**). Anticoagulation in this case may be controversial. Although anticoagulation is necessary after ALI treatment to reduce the risk of ALI recurrence, this patient also had a risk of microembolization, which developed in the right toes. Therefore, we limited anticoagulation to immediately after thrombectomy. Additionally, anticoagulation therapy is essential in patients with atrial fibrillation. However, this case involved ALI caused by AAA mural thrombosis; therefore, we determined that long-term anticoagulation after EVAR was not essential.

Thrombosis due to AAA is rare but fatal once developed.^1)^ A previous report regarding EVAR for AAA thrombosis has reported poor outcomes even after technical success.^4)^ These studies have suggested that poor extremity perfusion is associated with postoperative outcomes.^2–4)^ In this current case of Rutherford class IIb ALI, urgent revascularization could be performed. Several previous studies have reported AAA occlusion, which, despite being clinically relevant, differs from AAA-induced ALI. In such cases, earlier extremity reperfusion may be effective in obtaining good postoperative outcomes. Open repair is the first choice for elective AAA surgery^8)^; however, it might be better if AAA-induced ALI also follows the ALI treatment strategy.^6,7)^ Invasive open repair has a risk of poor extremity perfusion due to aortic clamping, bleeding, and hypotension. Maintaining extremity perfusion and sufficient anticoagulation is essential for ALI treatment,^6,7)^ and thus we thought EVAR might potentially have advantages in such cases.

## Conclusion

We demonstrate successful thrombectomy and EVAR for ALI due to AAA mural thrombus. The key to this successful outcome was earlier extremity reperfusion and less invasive aortic repair.
